# Efficient non-Hermitian wave-modulation protocol with a rapid parametric jump

**DOI:** 10.1515/nanoph-2024-0694

**Published:** 2025-02-14

**Authors:** Seung Han Shin, Yu Sung Choi, Yae Jun Kim, Jae Woong Yoon

**Affiliations:** Department of Physics, 26716 Hanyang University, Seoul, South Korea

**Keywords:** non-Hermitian Hamiltonians, exceptional points, adiabatic processes, waveguides, optical modulators

## Abstract

Non-Hermitian photonic wave modulators utilizing exceptional points (EPs) was previously proposed as a potential approach to realize high-performance and compact optical modulators. However, their practical implementation has been restricted by their substantial footprint size limit due to stringent adiabatic conditions near EPs. Here, we demonstrate a principle for efficient wave modulation through optimized parametric trajectories around an EP. By employing rapid encirclement or parametric jump over the EP, we achieve an extinction ratio of 43 dB within a total device length of 15 coupling-length unit. Importantly, we show that adiabatic processes around the EP in the initial stage of the entire evolution can be replaced by an instantaneous parametric jump without compromising the switching performance in stark contrast to the conventional wisdom from the standard adiabaticity.

## 1 Introduction

Exceptional point (EP) is a parametric square root singularity in a non-Hermitian (NH) system where multiple eigenstates coalesce into a singular state [1]. EP is observed for variety of different physical systems [2], [3], [4], [5] and associated with anomalous phenomena including spontaneous symmetry-breaking phase transition [6], [7], [8], unidirectional light propagation [9], [10], direction-selective lasing [11], [12], [13], [14], and zero-group-speed effect [15]. Photonic waveguide is one of the most effective photonic structures for utilizing EP-related effects [16], [17], [18], [19], [20], [21], [22], [23], [24], [25], [26], [27] because of its capability to create intricate non-Hermitian Hamiltonians in remarkably simple structures.

Within this context, adiabatic processes in the vicinity of an EP are of special interest. An EP forms a characteristic complex-square-root geometry that enables time-asymmetric guided-mode transfer while dynamically steering the systems condition around it [16], [17], [18], [19], [20], [21], [22], [23], [25]. Recent studies have revealed that the effect persists even for parametric loops not enclosing an EP [23], [26] and for fast parametric-steering processes beyond the adiabatic limit [25]. In another study, closely bypassing-EP processes were proposed as an efficient wave modulation and switching method [27]. In this proposal, indefinitely small change in the bypassing route results in a step-like abrupt change in the final state, thereby producing an efficient modal-switching mechanism. In these examples, corresponding waveguide structures are designed based on time-varying non-Hermitian Hamiltonians within the adiabatic limit and subsequently substantially large footprint length is often unavoidable, restricting their potential for applications in practice.

In this paper, we propose an improved parametric passage protocol for faster EP-bypass wave-modulation effect that offers practical waveguide designs with remarkably shorter foot-print length. Our proposed protocol combines two separate effects of super-adiabatic state-evolution property for fast encircling-an-EP passages [25] and mode-switching action for EP-bypassing passages [27]. We obtain 43 dB extinction ratio in a remarkably short design with a length of only 15 coupling-length unit, which is significantly smaller than 100 in the previous approach [27]. We systematically analyse parametric passage profiles and progression speeds to establish optimal designs. We validate the proposed protocol through rigorous numerical simulation of an exemplary InGaAsP waveguide structure.

## 2 Results and discussion

### 2.1 Dynamics in two-level NH systems

The standard adiabatic theorem states that a dynamic state follows an instantaneous eigenstate, also referred to as adiabatic state, under the condition

(1) $$\frac{\left\langle n\left(t\right)\right|\dot{H}\left|m\left(t\right)\right\rangle }{{\left|\Delta \lambda \right|}^{2}}\ll 1.$$

Here, **H** represents a Hamiltonian, *n*(*t*) and *m*(*t*) denote instantaneous (adiabatic) eigenstates, and Δ*λ* represents the eigenvalue difference between |*n*(*t*)⟩ and |*m*(*t*)⟩. In contrast to Hermitian systems, this condition does not strictly apply to non-Hermitian systems because of their non-conserving nature represented by complex eigenvalue spectra. In particular, the non-conserving property appears in a specific way such that non-adiabatic transition is selectively enhanced towards a specific eigenstate with the least imaginary eigenvalue [16], [17], [18], [20], [21], [22], [23].

Such properties are comprehensively described by a bi-orthogonal-basis treatment for generic non-orthogonal eigensystem [28]. The biorthogonal basis set consists of left eigenstates, *i.e.*, ⟨*χ*_*n*_|**H** = ⟨*χ*_*n*_|*λ*_*n*_, and right eigenstates, *i.e.*, **H**|*ϕ*_*n*_⟩ = *λ*_*n*_|*ϕ*_*n*_⟩, such that they form a bi-orthonormal inner-product space with the relation ⟨*χ*_*n*_|*ϕ*_*n*_⟩ = *δ*_*nm*_. We take this treatment in our theoretical description hereafter.

We examine a binary NH system that evolves by the following equation of motion [17]

(2) $$\frac{d}{dt}\left[\begin{array}{c} c_{1}\\ c_{2} \end{array}\right]=i\left[\begin{array}{cc} \lambda_{1} & -ig_{12}\\ -ig_{21} & \lambda_{2} \end{array}\right]\left[\begin{array}{c} c_{1}\\ c_{2} \end{array}\right],$$

where *c*_*n*_ denotes adiabatic-state amplitude, *λ*_*n*_ represents instantaneous eigenvalue which is complex-valued in general, and *g*_*nm*_ = ⟨*χ*_*n*_|**Ḣ**|*ϕ*_*m*_⟩(*λ*_*n*_–*λ*_*m*_)^−1^ is non-adiabatic coupling potential. In this description, we use a convention for the state index *n* such that the amplifying adiabatic state takes *n* = 1 while *n* = 2 for the attenuating adiabatic state, implying Im(*λ*_1_) < Im(*λ*_2_).

We examine properties of probability ratio |*c*_1_/*c*_2_|^2^ in the slow and fast time-varying limits to get some basic insights. In the slow limit where the adiabatic condition in Eq. (1) is sufficiently satisfied and *g*_*nm*_ is negligible, the ratio follows

(3) c1(t)c2(t)2≈c1(0)c2(0)2⁡exp2Imλ2−λ1t.

As Im(*λ*_2_−*λ*_1_) > 0 in the exponent, the ratio amplifies with time and the amplifying state (*c*_1_) dominates the response of the system, regardless of the initial-state condition represented by |*c*_1_(0)|^2^/|*c*_2_(0)|^2^. Consequently, partial probability |*c*_1_(*t*)|^2^/[|*c*_1_(*t*)|^2^ + |*c*_2_(*t*)|^2^] that the system is found at the amplifying eigenstate tends to 100 % regardless of the initial condition.

In the very fast limit that |*g*_*nm*_| >> |*λ*_*l*_| in contrast, the effect of non-adiabatic coupling potential *g*_*nm*_ dominates the dynamics and Eq. (2) reduces to

(4) $$\frac{d}{dt}\left[\begin{array}{c} c_{1}\\ c_{2} \end{array}\right]\approx \left[\begin{array}{cc} 0 & g_{12}\\ g_{21} & 0 \end{array}\right]\left[\begin{array}{c} c_{1}\\ c_{2} \end{array}\right].$$

Equation (4) yields a set of approximate solutions

(5) $$c_{1}\left(t\right)\approx c_{1}^{+}e^{+gt}+c_{1}^{-}e^{-gt},$$

(6) $$c_{2}\left(t\right)\approx c_{2}^{+}e^{+gt}+c_{2}^{-}e^{-gt},$$

with *g* = (*g*_12_ *g*_21_)^1/2^ and the coefficients

(7) $$c_{1}^{\pm }=\frac{1}{2}\left[c_{1}\left(0\right)\pm \sqrt{\frac{g_{12}}{g_{21}}}c_{2}\left(0\right)\right],$$

(8) $$c_{2}^{\pm }=\frac{1}{2}\left[c_{2}\left(0\right)\pm \sqrt{\frac{g_{21}}{g_{12}}}c_{1}\left(0\right)\right].$$

From Eqs. (5)–(8), the probability ratio converges to

(9) $$\begin{array}{ll} {\left|\frac{c_{1}\left(t\to \infty \right)}{c_{2}\left(t\to \infty \right)}\right|}^{2} & \approx \left|\frac{g_{12}}{g_{21}}\right|=1. \end{array}$$

Therefore, the dynamic state becomes a superposition of the amplifying and attenuating eigenstates with equal probability regardless of the initial condition.

In intermediate cases between the slow and fast limits, neither *g*_*nm*_ nor *λ*_*i*_ is negligible and the description is subsequently complicated with their respective effects. Nevertheless, we can intuitively infer the system’s overall responses as ending up in the amplifying adiabatic state regardless of initial state conditions. Although Eq. (9) predicts the final 50:50 superposition of the amplifying and attenuating adiabatic states in the fast limit, the exponential effect of non-zero imaginary eigenvalue splitting Im(*λ*_2_−*λ*_1_) > 0 in an intermediate case dominates the system’s response and the dynamic state relaxes at the amplifying adiabatic state according to Eq. (3). This implies that the amplifying adiabatic state is always the system’s preferred final state unless the evolution passage time is too short in the limit *t* << Im(*λ*_2_−*λ*_1_)^−1^. This is an essential NH property leading to the fast mode-switching protocol that we propose in the next section.

### 2.2 Mode-switching action at an EP

We consider a two-level NH system described by the following NH Hamiltonian and the Schrödinger-type equation

(10) $$H\left(\tau \right)=\left[\begin{array}{cc} p\left(\tau \right)+iq\left(\tau \right) & 1\\ 1 & -p\left(\tau \right)-iq\left(\tau \right) \end{array}\right],$$

(11) $$\frac{d}{d\tau }\left|\psi \right\rangle =iH\left(\tau \right)\left|\psi \right\rangle .$$

Here, *τ* represents relative time to the interstate coupling period. *p*(*τ*) and *q*(*τ*) are normalized real and imaginary energy splitting between the two basis states, respectively, and treated as key parameters that govern the time-varying property of the system. Note that **H** is PT-symmetric along *q* axis at *p* = 0 and involves two PT-symmetric EPs at (*p*, *q*) = (0, ±1).

**Figure 1:**
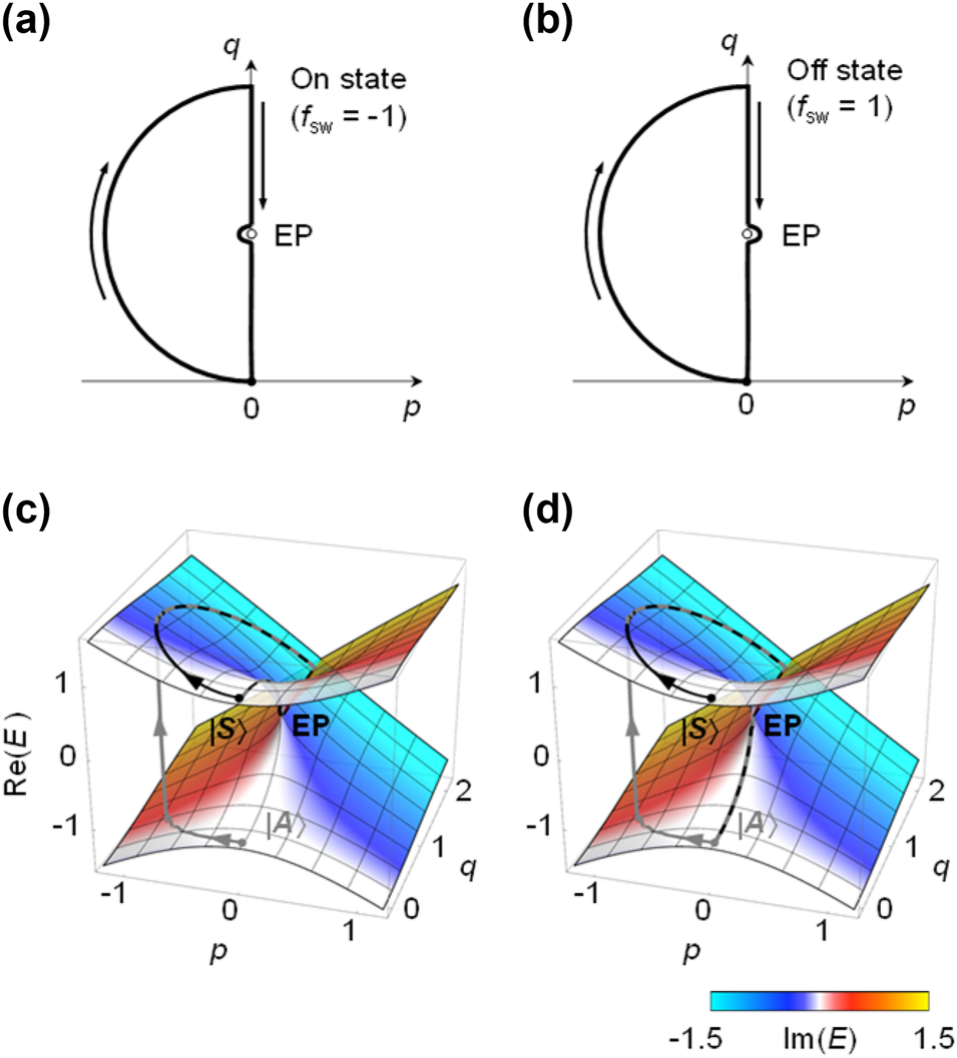
Dynamic state encircling an EP on *p*-*q* plane. (a, b) On state and Off state paths on the *p*-*q* plane, consisting of a detour path section and an EP-bypass path section. The On state path does not encircle the EP, while the Off state path does. (c, d) Real eigenvalue surfaces of Hamiltonian in Eq. (10), with color indicating the imaginary eigenvalue, plotted alongside with ⟨**H**⟩ of the dynamic state following the route in (a) and (b). The dynamic states with symmetric and anti-symmetric initial states are plotted as black and gray lines, respectively.

The switching parametric routes that propose here are shown in Figure 1a and b. Each of them consists of two sections – a fast detour-path section that brings the system to the large imaginary-eigenvalue-splitting region and a slow adiabatic-path section that closely bypass either one of the two EPs. In the detour path, the dynamic state is prepared to relax at the amplifying adiabatic state regardless of the selected initial state as fast as possible. In the subsequent adiabatic path, the dynamic state adiabatically evolves into the system’s preferred final state along the amplifying adiabatic state. The system’s preferred final state is switched between two orthogonal eigenstates at the parametric destination at (*p*, *q*) = (0, 0) depending on the EP-bypass direction. The preferred final state is the symmetric state |*S*⟩ for the left-side bypass or the anti-symmetric state |*A*⟩ for the right-side bypass as shown in Figure 1c and d, respectively.

For the sake of convenience in the numerical analyses, we assume a circular detour path from (*p*, *q*) = (0, 0) to (0, 2) and a Gaussian-step bypass profile along the PT-symmetric line from (0, 2) to (0, 0). The Gaussian-step profile is defined in the same manner as our previous study in [27] by

(12) $$p=f_{\text{sw}}B_{0}\theta \left(2\frac{q-q_{1}}{w}\right)\theta \left(2\frac{q_{2}-q}{w}\right).$$

Here, *θ* (*x*) is a Gaussian unit-step function

(13) $$\theta \left(x\right)=\frac{1}{\sqrt{\pi }}\overset{x}{\underset{-\infty }{\int }}\exp\left[-\xi^{2}\right]d\xi ,$$

with *B*_0_ = 0.01, *w* = 0.01, *q*_1_ = 0.99, and *q*_2_ = 1.01. Switching factor *f*_sw_ continuously determines the bypass magnitude and direction for given reference bypass profile for *f*_sw_ = 1. For example, if *f*_sw_ = +0.1 or −0.1, the adiabatic path bypasses the EP on the right (+) or on the left (−) through a route 10-times closer than the reference route.

Typical dynamic-state evolution and the consequent mode-switching behaviour are shown in Figure 1c and d. They show the dynamic-state passages of energy expectation value ⟨**H**⟩ on the real-eigenvalue surface with its skin colour indicating the imaginary eigenvalue. We confirm in this simulation that the desired mode-switching function is precisely operative. In Figure 1c for the parametric route (*f*_sw_ = −1) in Figure 1a, the final state is |*S*⟩ regardless of the initial-state selection. In contrast, the final state is |*A*⟩ regardless of the initial-state selection as shown in Figure 1d for the parametric route (*f*_sw_ = +1) in Figure 1b. Therefore, the proposed loop-form protocol properly enables the mode-switching action that was originally proposed in [27] with a linear return-path protocol. In addition, the proposed loop-form protocol remarkably outperforms the previous linear return-path protocol in the speed of the switching action as we show in the next section.

### 2.3 Progression speed and route optimization

Simultaneous optimization of parametric route and progression speed profile is required to achieve high switching performance within a shorter process time. Mode-switching performance depends on the modal purity of the final dynamic state with respect to the final amplifying adiabatic state. According to our theoretical argument in the previous sections, the high modal purity is obtained when the process along the bypass segment is in the slow adiabatic limit. If the evolution is too fast beyond the adiabatic limit in the vicinity of the EP or on the PT-symmetric line towards the end of the process, the final state end up in a certain binary superposition of |*S*⟩ and |*A*⟩ because of non-adiabatic transitions in the absence of the NH exponential relaxation mechanism. Note that the exponential relaxation mechanism is activated with the imaginary eigenvalue splitting Im(*λ*_2_−*λ*_1_) which is zero along the PT-symmetric line below the EP, *i.e.*, *p* = 0 line over 0 ≤ *q* < 1 domain. This implies that even if the EP is bypassed adiabatically, failing to maintain adiabaticity along the PT-symmetric line causes an impure final state and associated interference between |*S*⟩ and |*A*⟩, which causes characteristic fringes and associated instability as reported in [27].

For the detour section in contrast, we do not have to operate the process strictly in the adiabatic limit because the imaginary eigenvalue splitting is large and the exponential relaxation mechanism is strongly activated. Therefore, an ideal progression-speed profile should be obtained with certain numerical optimization methods that accommodate the two distinguished parts of fast pace on the detour section and slow pace on the EP-bypass section.

Taking these requisites into account in our trial optimization, we define the progression speed as *v* = [(*dp*/*dτ*)^2^+(*dq*/*dτ*)^2^]^1/2^ that follows a prescribed rule by

(14) $$v=v_{\text{avg}}N_{v}{\left|\Delta \lambda \right|}^{J},$$

where *v*_avg_ is average speed *L*/*T* with *L* and *T* denoting the total evolution distance on *p*-*q* plane and total time, respectively, *N*_*v*_ is a scale constant, Δ*λ* represents the eigenvalue difference, and *J* is a speed-profile order that we use as a main optimization parameter. Equation (14) controls *v* such that the progression slows down near the EP while being accelerated elsewhere with respect to *v*_avg_. Increasing *J* results in the deceleration and acceleration at a greater magnitude.

The trial optimization is formulated in the form of Eq. (14) because the adiabatic theorem states that transitions between eigenstates in a time-varying Hamiltonian are described by the left-hand side of Eq. (1). Therefore, the rates of change for the parameters *p* and *q* that control the variation of the Hamiltonian are set proportional to the energy difference at each point along the route. Nevetheless, we note that Eq. (14) as a reasonable trial does not always guarantee a global optimum. Therefore, other rules for the progression speed profile or purely numerical metaheuristic optimization techniques can be applied for improved performance. It is open for further follow-up study.

Considering Eq. (14) in relation to the standard adiabatic condition in Eq. (1), the speed-profile order must be optimized at *J* = 2 for sufficiently large total-evolution time *T*. However, we demand sufficiently strong switching effect for *T* as small as possible. In such cases, the progression may not be within the adiabatic limit for some or all parts of the entire process and the non-adiabatic transition exists in general. In the presence of the non-adiabatic transition for small *T*, the optimal *J* might be significantly deviated from 2 and thereby it is subject to a numerical optimization.

*J* is optimized for maximal switching-extinction ratio (ER) at the final dynamic state |*ψ*_*f*_ ⟩ = |*ψ*_*f*_ (*t* = *T*)⟩. The ER is defined by

(15) $$r_{\text{ext}}=\frac{{\left|\left\langle S\left|\right.\psi_{f}\left(f_{sw}=-\alpha \right)\right\rangle \right|}^{2}}{{\left|\left\langle S\left|\right.\psi_{f}\left(f_{sw}=+\alpha \right)\right\rangle \right|}^{2}}.$$

Here, *f*_sw_ = −*α* is the switching-factor condition for the On state and *f*_sw_ = +*α* is for the Off state. In this On/Off state description, we assume that |*ψ*_*f*_ ⟩ = |*S*⟩ or |*A*⟩ is the ideal On or Off state, respectively. The ideal On and Off states yields *r*_ext_ = ∞. In non-ideal cases that we have to play with, |*ψ*_*f*_ ⟩ is a superposition of |*S*⟩ and |*A*⟩ in general and *r*_ext_ takes a certain finite value.

**Figure 2:**
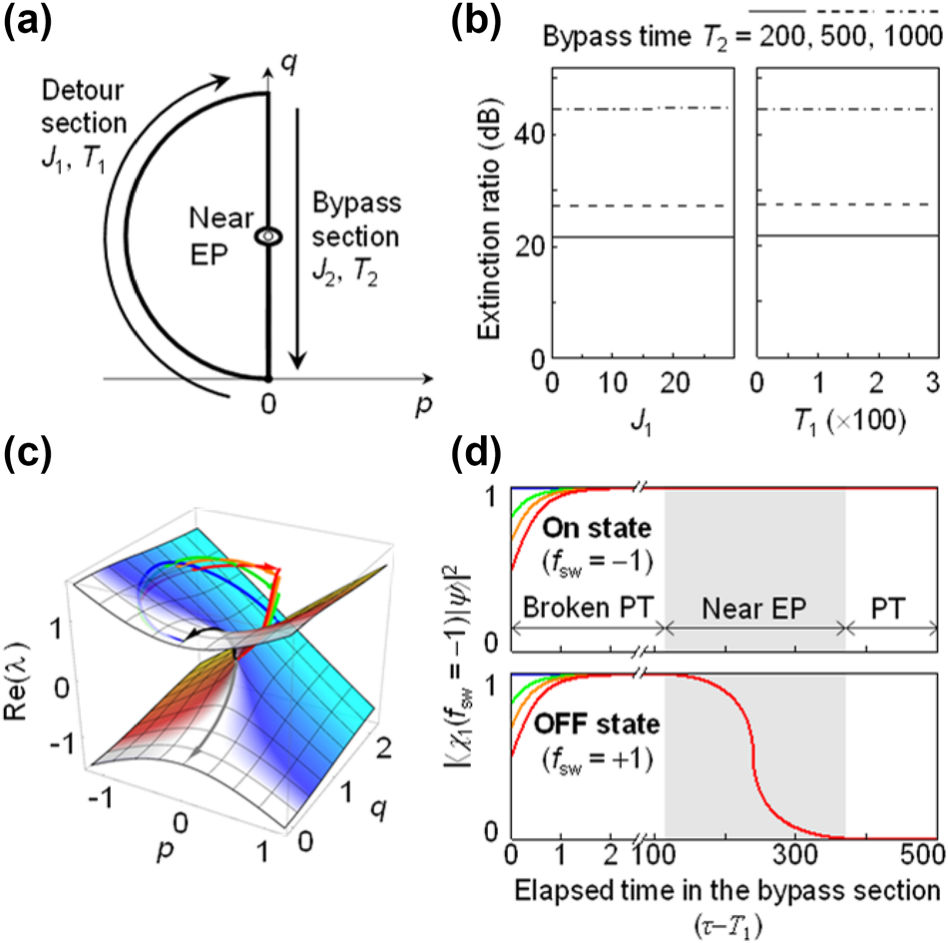
Effect of progression speed on switching ER. (a) On/Off state routes on the *p*-*q* plane under optimization. Progression speeds on detour and EP-bypass path sections are optimized using {*J*_1_, *T*_1_} and {*J*_2_, *T*_2_}, respectively. (b) ER for *J*_1_ ranging from 0 to 30 with *T*_1_ = 25 (left pannel) and for *T*_1_ ranging from 0 to 300 with *J*_1_ = 1 (right pannel). Both (a) and (b) adopt three distinct bypass processes with *T*_2_ = 200, 500, 1,000 and *J*_2_ = 3.3. (c) ⟨**H**⟩ of dynamic states on eigenvalue surfaces with *T*_1_ = 0.01, 0.5, 1, 10 and *J*_1_ = 3.3, shown as red, orange, green, and blue lines, respectively. In all cases, the bypass process uses *T*_2_ = 500 and *J*_2_ = 3.3. (d) Temporal evolution of on-state amplifying eigenstate probability of dynamic states following On state (upper pannel) and Off state (lower pannel). The colored lines represent identical {*J*_1_, *T*_1_, *J*_2_, *T*_2_} conditions as (c).

We analyse the effect of changes in *J* and parametric route on the ER for comprehensive optimization. We assign independent *J* and *T* for the detour and EP-bypass sections – *J*_1_ and *T*_1_ for the detour section; *J*_2_ and *T*_2_ for the EP-bypass section, as shown in Figure 2a. First, we investigate impact of *J* and *T* for the fixed process route used in Figure 1. We examined the effects of all four parameters {*J*_1_, *T*_1_, *J*_2_, *T*_2_} for *f*_*sw*_ = ±1 and found out that {*J*_1_, *T*_1_} change do not make any substantial difference. In Figure 2b, we show the ER dependences on *J*_1_ and *T*_1_ at fixed respectively. The ER remains constant as *J*_1_ and *T*_1_ change. In contrast, the ER sensitively takes different values as *T*_2_ changes from 200 to 1,000. Therefore, we can rule out {*J*_1_, *T*_1_} from the optimization parameters and fix them at a certain appropriate point. Remarkably, the appropriate point should be *T*_1_ = 0 because the ER is still constant threat. This implies that the final state is not affected by the detour-section condition at all and thereby we can remove the entire detour section from the process for obtaining desired final state and mode-switching performance. This is rather surprising because a sudden jump to the starting point of the EP-bypass section is allowed without any restriction.

This intriguing property is available in general as far as the starting point of the EP-bypass section is at any point in domains with sufficiently large imaginary-eigenvalue splitting around the broken-PT-symmetry phase region. In Figure 2c, we show the dynamic expectation-value ⟨**H**⟩ passages for *T*_1_ = 0.01, 0.5, 1, 10 on the real-eigenvalue Riemann sheet. Even though different *T*_1_ values result in different ⟨**H**⟩ at the end of the semi-circular detour section but the final ⟨**H**⟩ at the end of the EP-bypass section along the PT-symmetry line (*p* = 0) is fixed at the upper eigenvalue, implying that |*ψ*_*f*_ ⟩ = |*S*⟩ regardless of the detour-section condition.

The reason for this effect is that the dynamic state for the EP-bypass section always converges to the gain mode regardless of the evolution history of the state in the detour section. In Figure 2d, we show how partial probability changes during the process on the EP-bypass section. The vertical axis indicates the probability of finding the dynamic state in the gain-mode adiabatic-state ⟨*χ*_1_| for *f*_sw_ = −1 (On state). Note that ⟨*χ*_1_| for *f*_sw_ = −1 and +1 are identical to each other at the initial stage in the broken PT-symmetry phase and are bifurcated to their respective preferred states during the close bypass in the near-EP region. The partial probability profiles for *T*_1_ = 0.01, 0.5, 1, 10 are at different points at the beginning (*τ*−*T*_1_ = 0) of the EP-bypass section but it tends to 100 % in the broken PT-symmetry phase region. Afterwards, the probability stays at 100 % for the On state while switches to 0 % for the Off state regardless of the outcome of the process during the detour section. This is a typical non-Hermitian property in the broken-PT-symmetry phase where large imaginary-eigenvalue spitting induces the exponential relaxation of a dynamic state to the amplifying adiabatic state. Therefore, an optimal parametric route for the desired switching effect can be comprised of an initial jump towards a point in the broken-PT-symmetry phase region and a subsequent EP-bypass section that secures the non-Hermitian exponential relaxation mechanism.

In further consideration, we see in Figure 2d that the relative process time taken to complete the exponential relaxation to the amplifying adiabatic state is around 2, which is merely 0.4 % of the EP-bypass section time duration *T*_2_ = 500. It suggests an additional room to reduce the overall process time without significantly compromising the ER. One approach is to accelerate the progression speed in the broken PT-symmetric region. For example in Figure 2d, the time domain between 2 and 100 can be removed without any degradation of the ER.

**Figure 3:**
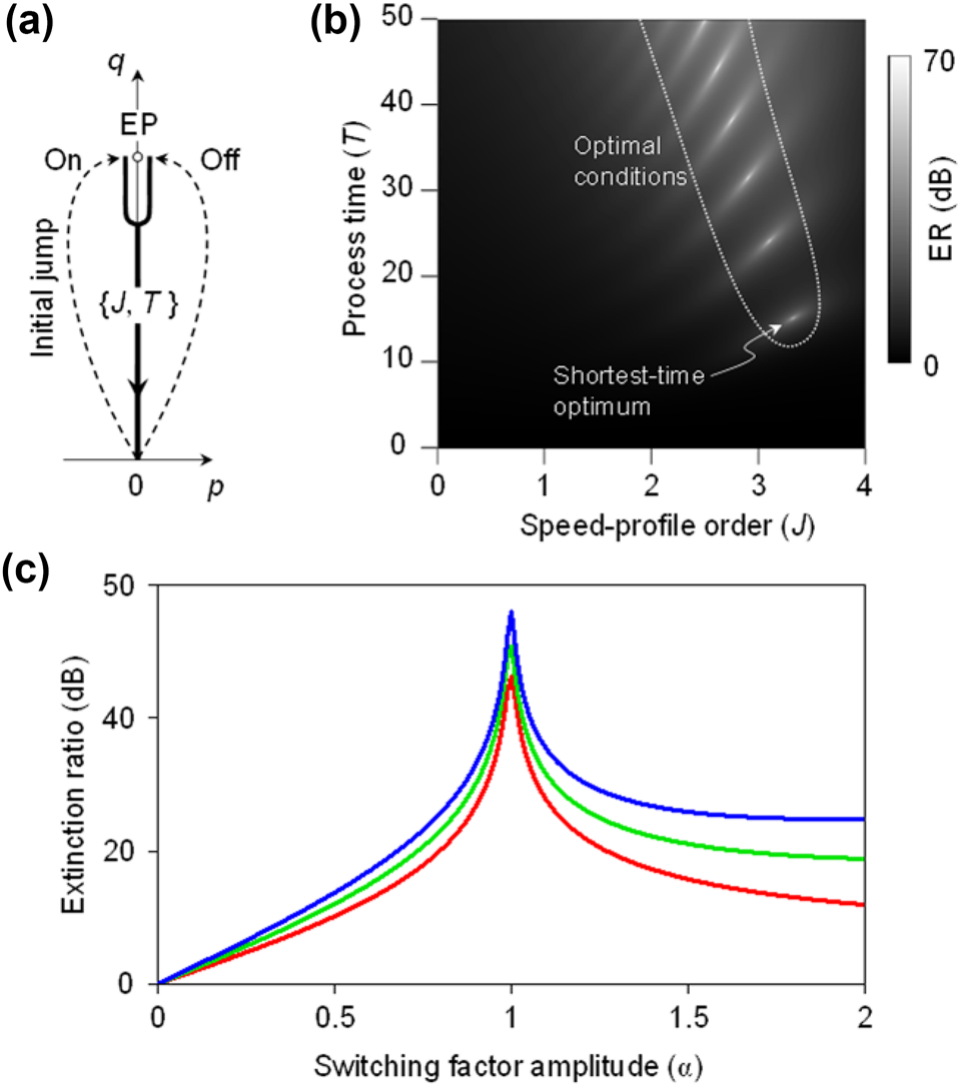
Optimized parametric route and ER. (a) Black solid line representing optimized parametric route for the On state and Off state. Detour section is replaced with a parametric jump. (b) Density plot of the ER as a function of *J* and *T* over 0 < *J* < 4 and 0 < *T* < 50. Shortest-time optimum is achieved at {*J*, *T*} = {3.3, 15} with ER of 43 dB. (c) ER as a function of *f*_sw_ amplitude. The red, green, and blue lines represent the first, second, and third shortest-time optima shown in (b), respectively.

Another is to start the EP-bypass section at a point close to the EP and secure a transient time thereat for the exponential relaxation to be complete. We adopt the latter in our trial parametric route design in Figure 3. The optimized parametric route begins with an initial jump to (*p*, *q*) = (−0.01, 1) for the On state or (+0.01, 1) for the Off state, as indicated in Figure 3a. The calculated ER map on *J*-*T* plane is provided in Figure 3b. The impact of the detour section parameters {*J*_1_, *T*_1_} on the final state is also analysed for this optimized route (see Supplementary Material, S1).

As we do not include the detour section anymore, we denote *J*_2_ and *T*_2_ simply by *J* and *T*, respectively, hereafter. The route-optimization results reveal that the optimum is achieved at a significantly shorter *T* compared to the route considered in Figure 2 by minimizing the evolution time in the region with imaginary eigenvalue splitting. It shows multiple optimal conditions where the ER is locally maximal. The local optimal point at {*J*, *T*} = {3.3, 15} is the most favourable condition as total evolution time is shortest. The ER value at this condition is 43 dB. In the previous approach outlined in [27], similar ER was obtained for remarkably long evolution time *T* > 100. In Figure 3c, we show dependence of ER on switching factor amplitude *α* for the three shortest-time optima in Figure 3b. At *α* = 1 where the condition in Figure 3b is calculated, all three cases exhibit high ER peaks with small differences in their peak values. For longer process-time conditions, the ER increases more rapidly as *α* rises from 0 to 1. For *α* > 1, the ER converges to higher values as the process time increases beyond the standard adiabatic limit.

### 2.4 Application to optical modulators

**Figure 4:**
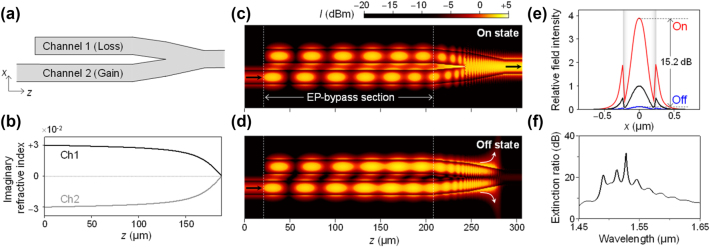
Coupled waveguide modulator configuration and BPM simulation. (a) Schematic of an EP-bypass waveguide modulator with attenuating (channel 1) and amplifying (channel 2) waveguides. (b) Imaginary refractive index profile of waveguide channel 1 and 2. (c, d) BPM simulation of a coupled waveguide modulator with refractive index modulation applied to channel 1 for the On state and without refractive index modulation for the Off state, operating at a wavelength of 1,550 nm. (e) Comparision of output electric field intensity in the On state (red) and Off state (blue) with the input (black). The shaded region represents the InGaAsP core, while the rest corresponds to the SiO_2_ cladding. (f) ER as a function of free space wavelength ranging from 1.45 to 1.65 μm.

We numerically demonstrate a coupled waveguide structure that enables our improved state-switching protocol suggested in the previous sections. The standard coupled-mode theory of a binary coupled-waveguide system is mathematically identical to Eqs. (10) and (11) if we eliminate dynamic-phase part in the wave function by taking the rotating-wave approximation. We assume InGaAsP core and SiO_2_ clad waveguide structures as an active integrated optics platform where electro-optic index control, gain, and losses can be conveniently included. A potential NH waveguide structure here consists of two coupled channel waveguides as shown in Figure 4a. The input optical signal is incident at Channel 2 with gain, immediately couples to Channel 1 with loss at the input terminal where the initial parametric jump happens, undergoes the desired EP-bypass process, and finally transmits through the Y-branch at the output terminal. Each channel waveguide features a 400-nm-wide and 250-nm-thick InGaAsP core designed to operate at a wavelength of 1,550 nm. The gap between the two waveguide cores is 200-nm-wide.

The EP-bypass process is created by having imaginary index profiles in the channels as shown in Figure 3b. Although we assume direct imaginary refractive index variation of the waveguide core and this may seem impractical, complex effective-index variation approach based on waveguide-width and radiation-loss tuning is available for practical device realization as demonstrated in [16], [17]. This gain-loss profile is optimized under the conditions (*p*, *q*) = (±0.066, 1), *T* = 13, and *J* = 4.

The state-switching between the On and Off states is induced by a dynamic index tuning by Δ*n* = 0.004 at Channel 1. The assumed magnitude of Δ*n* can be obtained by a thermo-optic control with temperature difference 20 K or possibly by free-carrier-effect-based electro-optic controls [29].

We explain the detailed operation mechanism of this waveguide design in connection with the NH-Hamiltonian model described in the previous sections. The input wave is initially injected exclusively into the amplifying waveguide, establishing the initial state (0, 1). Within the coupled waveguide region, the two waveguides are separated by a 200 nm gap. Comparison with Eq. (10) shows that when propagation constant of waveguide Channel 1 is smaller than that of Channel 2 (*β*_1_ < *β*_2_), *p*(*τ*) becomes negative, causing the system to bypass the EP on the left and return to the symmetric state. Conversely, when *β*_1_ > *β*_2_, *p*(*τ*) becomes positive, the system bypasses EP to the right and reaching the anti-symmetric state. Thus, we can achieve switching centered at *p* = 0 by applying a slight refractive index offset to Channel 1, which adjusts *β*_1_ between two detuned values at *β*_2_ − Δ*β* and *β*_2_ + Δ*β* for the On and Off states. This control mechanism in our design is realized by slightly reducing the Channel 1 core width to 399 nm which yields *p* = Δ*β*/κ = 0.066.

We use the beam propagation method for numerical simulation of the design and the results are shown in Figure 4c and d. We confirm that the transmission at the output terminal is almost free for the On state and forbidden for the Off state as desired. Additionally, nearly identical switching effects occur even with an imaginary refractive index offset ranging from −0.01 to 0.01 (see Supplementary Material, S2). The intensity distributions for the On and Off states at the output terminal facet are shown in Figure 4e. The ER based on the output intensity distribution is 15.2 dB. Furthermore, an ER exceeding 10 dB is achieved across a broad wavelength range from 1.48 μm to 1.62 μm as provided in Figure 4f. Therefore, our proposed approach is confirmed in the rigorous numerical calculation.

In comparison, we consider a Mach–Zehnder interferometer (MZI) structure as a primary competing technology for on-chip optical modulators and switches. Assuming an identical waveguide structure and refractive index modulation, achieving mode conversion between two orthogonal modes in a MZI modulator requires a π-phase shift, necessitating a refractive index modulation region of over 200 µm. In contrast, our proposed NH-modulator approach enables such a mode conversion at a slightly shorter device length which might be further reduced with more advanced optimization methods for the progression-speed profile *v*(*τ*) as discussed in Section 2.3. In addition, the proposed, non-interferometric NH modulator enables broadband modulation at 10 dB extinction ratio over a wide range from 1.48 to 1.62 µm in contrast to the strictly single-wavelength operation for the corresponding MZI modulator. In spite of such advantages, the NH modulator poses challenges for practical device implementation because it involves independent gain and loss control, which requires extensive further experimental study.

In consideration of experimental realization, the changes in *p* and *q* correspond to variations in the real and imaginary effective indices of the guided mode, respectively. Therefore, certain appropriate structural variations that adjust the complex effective index distribution should be included in the design. Taking this into account in our trial design in Figure 4, the structure only involves the imaginary effective-index variation at magnitude of 3 × 10^−2^ refractive index unit (RIU). Since this value is merely 1.4 × 10^−2^ of reference effective index 2.1 RIU for the operative guided mode and thereby corresponding structural variation for the desired parametric evolution does not involve and abrupt structural change.

For example, Si-photonic waveguide structure for encircling-an-EP processes on a parametric domain in the similar scale to our case was demonstrated in [16]. Therein, the waveguide width and inter-waveguide spacing were used to control the complex effective index. In their case, the magnitudes of the width and spacing changes are less than 5 nm per 1 μm propagation, which is not abrupt structural change at all as far as fabrication is concerned. If we take the approach in [16] for our case in Figure 4, magnitude of the inter-waveguide spacing change is less than 30 nm per 1 μm propagation, which is still favorable for real fabrication.

In addition, if the parametric jump protocol proposed here is applied to the waveguide structure in the previous study [27], the y-branch before the EP-bypass region can be eliminated and the first half of the EP-bypass region where transition from the origin to the EP occur can be additionally removed. Such supplement structure elimination remarkably reduces the footprint length the entire device structure from 300 to 150 µm.

## 3 Conclusions

We demonstrate a novel principle for compact NH wave modulation through optimized parametric route near an EP. By systematically analysing the effects of parametric evolution speed and trajectory profiles, we establish that proper optimization of bypass processes enables significant simplification of parametric evolution protocol and subsequent device miniaturization without compromising switching performance. In particular, the adiabatic detour process preceding EP bypass that seems necessary in the conventional approaches can be replaced by a sudden jump process without affecting the final state in general. This is possible because the broken PT-symmetric line with strong imaginary eigenvalue splitting inherently drives transition to the amplifying eigenstate regardless of the dynamic state at the turning point. This fundamental property allows substantial reduction of device footprint size.

Our theoretical analysis and numerical simulations reveal that an ER of 43 dB is achieved with a total device length of just 15 coupling-period unit through the initial parametric jump. This represents a significant advancement in device miniaturization compared to conventional EP-based modulators that typically require stringent adiabatic conditions. The proposed approach fundamentally differs from previous implementations by strategically optimizing both the parametric trajectory and evolution speed.

Implementation in an InGaAsP-based coupled waveguide platform demonstrates the practical feasibility of our approach. The proposed coupled waveguide modulator configuration achieves an ER of 15.2 dB within the NH waveguide length of 188 μm for a refractive-index tuning amplitude of 0.004. This result exemplifies possibility for compact EP-based photonic devices in optical signal processing where device footprint and switching performance are critical considerations.

Looking forward, our findings provide a foundation for further optimization of EP-based photonic devices. The demonstrated principles of rapid EP encirclement and parametric jump could be extended to other non-Hermitian photonic systems, potentially enabling new classes of compact optical devices. Further investigation of dynamic properties near EPs and optimization of control schemes may yield additional performance improvements for next-generation optical communication systems.

## Supplementary Material

Supplementary Material Details
