# Supplementary material for: Efficient non-Hermitian wave-modulation protocol with a rapid parametric jump
Source: Nanophotonics. 2025 Feb 14;14(10):1635–43. doi: 10.1515/nanoph-2024-0694 (PMC12116274; doi:10.1515/nanoph-2024-0694)
Supplement: Supplementary file 1 — Supplementary Material Details [file j_nanoph-2024-0694_suppl_001.pdf]

Supplementary Materials

Seung Han Shin, Yu Sung Choi, Yae Jun Kim, Jae Woong Yoon*

Efficient non-Hermitian wave-modulation protocol with a rapid parametric jump

S1. The effect of detour process parameters on switching in the optimized route

Unlike the case discussed in Fig. 2, where the extinction ratio is entirely independent of the detour section, the route addressed in Fig. 3 shows that the extinction ratio is influenced by the detour path parameters under optimized conditions. This is because, during the route optimization process, most of the region with imaginary eigenvalue splitting is skipped, and the bypass route begins right next to the EP, where both the real and imaginary eigenvalue splittings are very small. As a result, the region where a transition to the amplifying eigenstate can occur becomes very short, leading to variations in the extinction ratio depending on T_1 and J_1, even for the same bypass process, as shown in Fig. S1. Fortunately, the changes in the extinction ratio are favorable. As seen in Fig. S1(a), the extinction ratio tends to increase as the detour section process time decreases. Additionally, as confirmed in Fig. S1(b), the speed profile order does not affect the extinction ratio in this region.

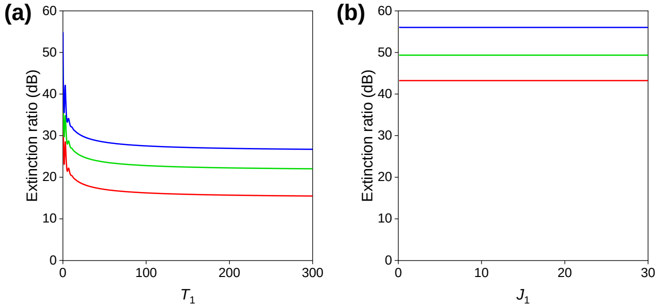

Figure S1. Detour process parameter dependence of extinction ratio in the optimized route. (a) Extinction ratio for T_1 ranging from0 to 300 while fixing J_1 at 1. (b) Extinction ratio for J_1 ranging from 0 to 30 while fixing T_1 at 0.1. Red, green, and blue line represent bypass section parameters $\{J_2, T_2\}$ set to shortest, 2nd-shortest, and 3rd-shortest time optimum obtained in Fig. 3(b) in the main text, respectively.

S2. Wave modulation with Imaginary refractive index offset

This is the result of BPM simulation performed with an imaginary refractive index offset applied to the identical waveguide structure presented in Section 2.4 of the main text. As shown in Fig. S1(a), an imaginary refractive index offset ranging from –0.01 to +0.01 does not affect the ER. Compared to the maximum absolute value of the imaginary refractive index (0.03) shown in Fig. 4(b) of the main text, this offset is relatively large, yet the proposed structure operates effectively. As an example, the simulation results for the On and Off states with imaginary refractive index offsets of +0.01 and –0.01 are shown in Figs. S1(b-e).

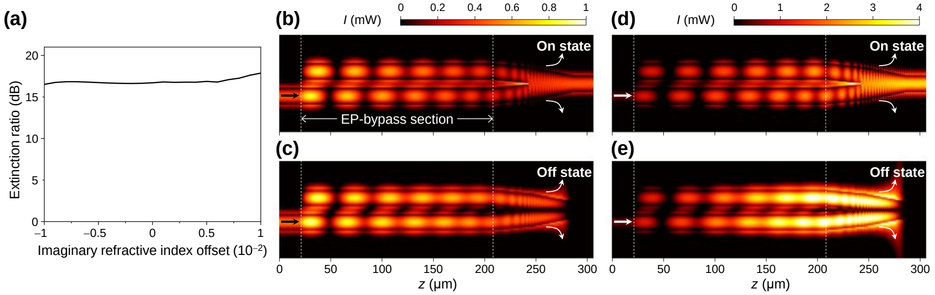

Figure S2. Wave modulation with Imaginary refractive index offset. (a) Extinction ratio achieved with imaginary refractive index offset ranging from –0.01 to 0.01. (b, c) BPM simulation of a coupled waveguide modulator for the On state and Off state with imaginary refractive index offset of +0.001. The color bar is shown above (b). (d, e) BPM simulation of a coupled waveguide modulator for the On state and Off state with imaginary refractive index offset of –0.001. The color bar is shown above (d).
